# Research on the spatial and temporal patterns of ozone concentration and population health effects in the Central Plains Urban Agglomeration from 2017 to 2020

**DOI:** 10.1371/journal.pone.0303274

**Published:** 2024-05-16

**Authors:** Jun Yan, Xinying Wang, Jiyuan Zhang, Zeyu Qin, Ting Wang, Qingzhi Tian, Shizhen Zhong

**Affiliations:** School of Geographic Sciences, Xinyang Normal University, Xinyang, China; Balochistan University of Information Technology Engineering and Management Sciences, PAKISTAN

## Abstract

Fine particulate matter (PM_2.5_) and near-surface ozone (O_3_) are the main atmospheric pollutants in China. Long-term exposure to high ozone concentrations adversely affects human health. It is of great significance to systematically analyze the spatiotemporal evolution mechanism and health effects of ozone pollution. Based on the ozone data of 91 monitoring stations in the Central Plains Urban Agglomeration from 2017 to 2020, the research used Kriging method and spatial autocorrelation analysis to investigate the spatiotemporal variations of ozone concentration. Additionally, the study assessed the health effects of ozone on the population using the population exposure risk model and exposure-response relationship model. The results indicated that: (1) The number of premature deaths caused by ozone pollution in the warm season were 37,053 at 95% confidence interval (95% CI: 28,190–45,930) in 2017, 37,685 (95% CI: 28,669–46,713) in 2018, and 37,655 (95% CI: 28,647–46,676) in 2019. (2) The ozone concentration of the Central Plains urban agglomeration showed a decreasing trend throughout the year and during the warm season from 2017 to 2020, there are two peaks monthly, one is June, and the other is September. (3) In the warm season, the high-risk areas of population exposure to ozone in the Central Plains Urban Agglomeration were mainly concentrated in urban areas. In general, the population exposure risk of the south is lower than that of the north. The number of premature deaths attributed to ozone concentration during the warm season has decreased, but some southern cities such as Xinyang and Zhumadian have also seen an increase in premature deaths. China has achieved significant results in air pollution control, but in areas with high ozone concentrations and high population density, the health burden caused by air pollution remains heavy, and stricter air pollution control policies need to be implemented.

## 1. Introduction

Ozone (O_3_) is a secondary air pollution attributed to the photochemical reactions between nitrogen oxides (NOx) and volatile organic compounds (VOCs) under solar ultraviolet radiation in the troposphere [1]. O_3_ is a very reactive gas and strong oxidant, which will strongly stimulate the respiratory tract and cause inflammation [2].

It will harm the resident’s public health if people might experience exposure to ozone concentrations on air pollution for a long term. With the acceleration of industrialization and urbanization, fine particulate matter (PM_2.5_) and near-surface ozone (O_3_) have become the main atmospheric pollutants in China. A large number of ozone precursor pollutants, such as NOx and VOCs, are discharged into the atmospheric environment, leading to the gradual increase of ozone concentration near the ground in China [3]. Atmospheric ozone pollution has a wide range of impacts and covers a wide range of people, which can cause huge population health risks and disease burden.

With the increasingly severe situation of ozone pollution, the harm it poses to human health has gradually attracted attention from the public [4].O_3_ poses potential hazards to human health and ecosystems.O_3_ exposure is closely associated with mortality, respiratory, and cardiovascular diseases. Acute or prolonged high-level exposure can lead to premature death, respiratory system diseases such as asthma and respiratory infections, cardiovascular diseases such as stroke and arrhythmia [5], as well as neurological disorders like autism in children and dementia in the elderly [6]. O_3_ can stimulate the respiratory system to produce a large amount of inflammatory cell hormones and accumulate toxic lipid oxidation products, ultimately leading to local chronic inflammation in the respiratory system. Furthermore, O_3_ can generate highly oxidative free radicals in the body, disrupt metabolism, induce lymphocyte chromosome abnormalities, damage the immune system, and accelerate aging [7]. Studies have shown that for every 10 μg/m^3^ increase in O_3_ concentration, there is a 0.48% increase in attributable mortality due to O_3_ exposure [8].

In the recent years, numerous studies have investigated the changes in O_3_ concentrations on a spatial and temporal scale. However, researchers are mostly concerned about the risk of human and vegetation exposure to surface O_3_ on the city scale, as well as the driving factors of O_3_ [9].For example, the spatiotemporal characteristics of surface O_3_ concentrations are explored in China [10],the United States [11], South Korea [12],India [13] and global scale. The population exposure risk of surface O_3_ is during the summer months in the United States. The O_3_-meteorology relationship showed spatiotemporal differences depending on the topographical and emission distribution characteristics of each area [14]. In terms of methods, they are mainly employed an exposure response model [15], spatial autocorrelation analysis [16], and concentrated in economically developed areas such as the Beijing Tianjin Hebei [17], the Pearl River Delta [18], and the Yangtze River Delta [19].

The Central Plains Urban Agglomeration (CPUA) is one of the important urban agglomerations in China. With rapid economic development, there is an increasing demand for a better environment from the public. However, in recent years, air pollution has become increasingly prominent in some cities within the CPUA, severely hindering its sustainable development. Currently, there is relatively little research on the ozone pollution exposure risk and its impact on public health in the CPUA from a spatial perspective, and the existing studies are based on earlier years and usually as part of nationwide research. Therefore, this study utilizes spatial analysis methods and population exposure risk index models based on the ozone concentration data from the national monitoring stations and population distribution grid data in the CPUA from 2017 to 2020. It aims to assess the spatiotemporal characteristics of ozone pollution and population exposure risk in the region and evaluate the impact of ozone pollution on public health based on exposure-response relationships.

## 2. Materials and methods

### 2.1. Study area

Central Plains Urban Agglomeration (CPUA) is located in the North China Plain, from110°15′E to 116°49′E and from 30°23′N to 37°47′N, and the lower reaches of the Yellow River. It belongs to the temperate monsoon climate, with an area of about 287,000 square kilometers and a total population of about 163.45 million, accounting for 11.54% of China’s population in 2021, and the GDP reached 8.87 trillion yuan, accounting for 7.75% of China’s GDP. The CPUA includes 30 cities in 5 provinces (Henan, Hebei, Shanxi, Shandong and Anhui) issued by the National Development and Reform Commission of the National Development and Reform Commission in 2016, with 13 prefecture-level cities such as Zhengzhou as the core development areas forming an urban agglomeration with close economic and social ties [20].It has a temperate monsoon climate with complex topographical conditions and the spatial-temporal variations and health impacts of O_3_ in the CPUA was investigated [21].

The CPUA plays a pivotal role in national agricultural production, which is population Human settlement and transportation hub in China, as well as an important area to undertake industrial transfer in overseas and eastern coastal areas. It also has a strong example of comprehensive strength, superior transportation location, complete urban system, excellent natural endowment, and profound cultural heritage.

### 2.2. Data source

The surface ozone concentration monitoring data used in this article comes from the real-time air quality monitoring platform provided by the China National Environmental Monitoring Centre (CNEMC), including hourly ozone concentration data from 129 monitoring stations in 30 cities from 2017 to 2020, provided by the National Urban Air Quality Real Time Release Platform of the CNEMC (http:106.37.208.233:20035/). Combining data integrity and the actual conditions of monitoring sites, ozone monitoring data from 91 monitoring stations were selected. The daily 8-h average standard for O_3_ was included in its ambient air quality standard (GB 3095–2012) in that year. Daily maximum 8-h average O_3_ concentrations (DMA8) data for 1st January, 2017 to 31st December, 2020 were obtained through 129 monitoring stations from CNEMC website which provides air quality data to the public. The daily data of ozone concentration is represented by the daily maximum 8-hour moving average ozone concentration (ozone-8h-max), and the monthly data of ozone concentration, cold season (from October to March of the following year), and warm season (from April to September) are represented by ozone-8h-max. According to technical regulation for ambient air quality assessment of China (on trial) (HJ 633–2013) published by the Ministry of Environmental Protection in 2013, the arithmetic mean of max and the annual data of ozone concentration use ozone-8h, which is equal to the 90^th^ percentile of DMA8.

**Fig 1:**
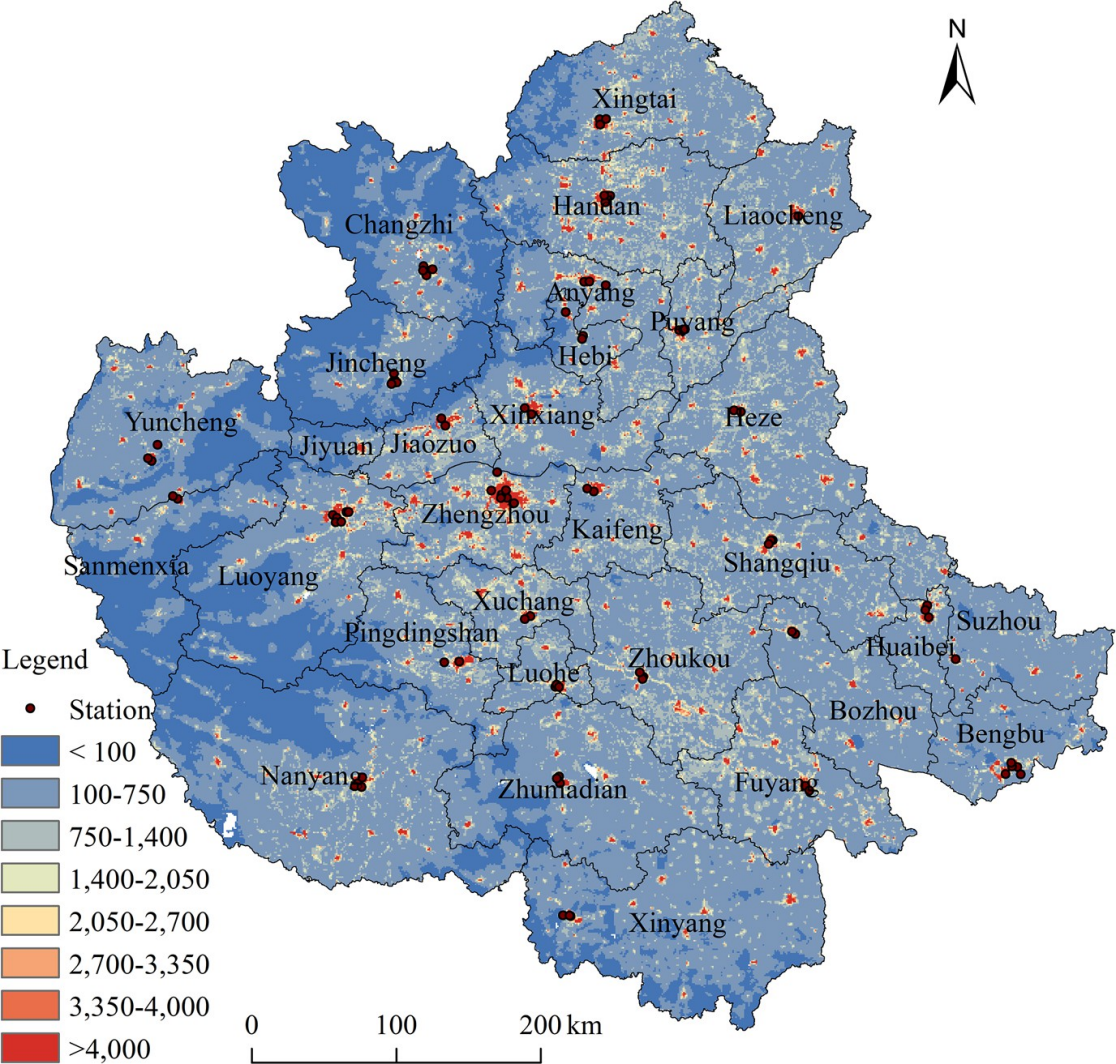
Spatial distribution of monitoring stations and population in CPUA.GS (2022)1873.

Spatial population data is derived from the 1km×1km population grid provided by WorldPop (https://hub.worldpop.org/), combined with the resident population data from various local government statistical yearbooks. The specific spatial distribution of sites and population distribution are shown in Fig 1.

### 2.3. Research method

#### 2.3.1. Spatial distribution feature

Spatial interpolation is an important method for visualizing the spatial distribution of ozone and studying its spatial characteristics. Commonly the spatial interpolation methods include Inverse Distance Weighted Interpolation (IDW), Completely Regularized Spline (CRS), and Ordinary Kriging (OK). According to relevant research, the Kriging method can achieve high simulation accuracy and reliability. Therefore, in this research, Ordinary Kriging is applied to interpolate the spatial distribution of ozone, in order to obtain ozone concentrations at the raster scale.

#### 2.3.2. Spatial autocorrelation analysis

Spatial autocorrelation refers to the potential interdependence of variables in the same distribution area [22], including global and local spatial autocorrelation. There are main two measures to analysis spatial autocorrelation, which are the global spatial autocorrelation index and the local spatial autocorrelation index [23]. Global spatial autocorrelation reflects the general trend of the spatial autocorrelation of raw data in the study area. In this study, global Moran’s I coefficient was used for global autocorrelation analysis [24].Moran’s I of global spatial autocorrelation is generally normalized based on the I value, and the value range of moran’s I is [–1,1], If I > 0, it demonstrates a positive spatial autocorrelation, which indicates that the frequency of ozone concentration in neighboring regions has a certain spatial interaction. If I < 0, it shows a negative spatial correlation, and If I = 0, it indicates that the frequency of ozone concentration is randomly distributed in space and there is no obvious rule. The equation of Moran’s I is as follows:

$$I=\frac{n\sum_{i=1}^{n}\sum_{j=1}^{n}w_{ij}(x_{i}-\bar{x})(x_{j}-\bar{x})}{(\sum_{i-1}^{n}\sum_{j=1}^{n}w_{ij})\sum_{i=1}^{n}{\left(x_{i}-\bar{x}\right)}^{2}}$$ (1)

$$\bar{x}=\frac{1}{n}\sum_{i=1}^{n}x_{i}$$

Where, *I* is the global Moran index; n is the number of spatial units (30 cities in this study), *w*_*ij*_ stands for the space weight matrix of the unit *i* and *j*, it reflects the spatial relationship between unit *i* and *j*, and it is defined as: if it is adjacent, *w*_*ij*_ = 1, otherwise, *w*_*ij*_ = 0; *x*_*i*_ and *x*_*j*_ are the change trend of O_3_ concentrations of spatial unit *i* and *j*, respectively, and $\bar{x}$ denotes the average O_3_ concentration of all the units.

A global spatial autocorrelation analysis is used to judge the aggregation trend of data, but the instability of the local space is not reflected [25]. To analyze spatial autocorrelation more accurately, we used a Hotspot analysis (Getis-Ord G_i_^*^) for the high-low clustering test, which can distinguish spatial clustering with statistical significance, such as high-value clustering (hot spots) and low-value clustering (cold spots). The hotspot clusters were mapped through Getis-Ord Gi* operation, using ArcGIS Spatial Statistics tools and fixed distance band [26]. Gi* represents the spatial autocorrelation that measures the correlation of a variable with itself through space [27]. GeoDa software is used for spatial autocorrelation analysis.The mathematical representation of Getis-Ord Gi* is expressed as follows:

$$G_{i}^{*}=\frac{\sum_{j=1}^{n}w_{ij}x_{j}-\bar{X}\sum_{j-1}^{n}w_{ij}}{s\sqrt{\frac{n\sum_{j=1}^{n}w_{ij}^{2}-{\left(\sum_{j=1}^{n}w_{ij}\right)}^{2}}{n-1}}}$$ (2)

where Gi* is the spatial autocorrelation of unit i over n unit, n is the number of spatial units, the term *x*_*i*_ characterizes the magnitude of the variable x at unit j over all n. $\bar{x}$ is the average O_3_ concentration of all the units and s is the standard deviation. The distribution of the Gi* statistic is normal when normality is observed in the underlying distribution of the variable x. The standardized Gi* can be associated with the statistical significance of a cluster. Positive and negative Gi* statistics with high absolute values correspond to clusters of O_3_ concentrations with high and low-value units, respectively.

#### 2.3.3. Population exposure risk model

There is a traditional population exposure risk model of EPC (E(exposure) = P (population density) × C (pollutant concentration), which can quantify the exposure intensity of population air pollution within a spatial unit to a certain extent. However, EPC has certain deficiencies in the prevention and control of regional air pollution health risk, such as the inability to directly distinguish the severity of population air pollution exposure within a certain spatial subunit relative to the overall spatial unit. For this reason, this study has constructed a relative risk assessment model for population exposure risk. The O_3_ concentrations population exposure risk index can evaluate the exposure status in each pixel of raster [28]. The formula is as follows:

$$R_{i}=\frac{p_{i}\times o_{3,i}}{\overset{n}{\underset{i=1}{\sum }}p_{i}\times \frac{o_{3,i}}{n}}$$ (3)

where i denotes the grid number; *R*_*i*_ represents the O_3_ concentrations population exposure risk index in grid i; *P*_*i*_ denotes the number of population within grid i; O_3,i_ denotes the O_3_ concentrations value within grid i; n is the sum of the number of grid nets in the study area.

#### 2.3.4. Exposure-response relationship model

Since existing epidemiological studies have mostly focused on the short-term health effects of ozone exposure and there is limited research on long-term health impacts, this study considers the accessibility and reliability of data and selects premature mortality caused by short-term ozone exposure as the health endpoint. According to the International Classification of Diseases, 10th edition, premature mortality is selected, including all-cause premature mortality (A00-R99), respiratory system disease premature mortality (J00-J99), and circulatory system disease premature mortality (I00-I99). The exposure-response coefficients for these endpoints are derived from a meta-analysis [29], the specific coefficients are shown in Table 1.

**Table 1:** Exposure response relationship coefficients for different health effect endpoints.

health effect endpoints	*β*%	95%CI
**all-cause premature mortality**	**0.40%**	**0.30%∼0.50%**
**respiratory system disease premature mortality**	**0.46%**	**0.23%∼0.70%**
**circulatory system disease premature mortality**	**0.50%**	**0.17%∼0.72%**

Additionally, studies have indicated that pollution during the warm season in China is more severe compared to other regions globally, and there is a significant association between warm-season ozone pollution and high mortality rates [30]. Therefore, the ozone evaluation indicator is the warm-season ozone concentration, and the threshold value is the warm-season ozone concentration value of 60 μg/m^3^ proposed by the World Health Organization (WHO).

In general, the occurrence of individual deaths or diseases is considered a rare event, which statistically follows a Poisson distribution. Therefore, this study uses a Poisson regression model to explain the exposure-response relationship of ozone concentration on premature mortality in the population [31]. The specific formulas are as follows:

$$\text{Y}=\frac{R-1}{R}Y_{0}POP$$ (4)

$$\text{R}=e^{\beta \left(x-x_{f}\right)}$$ (5)

Where Y represents the number of premature deaths caused by ozone in a particular year; Y_0_ is the baseline mortality rate for a specific disease in that year, obtained from the "China Health Statistics Yearbook" for that year; POP represents the population size in that year; R is the relative risk coefficient, which represents the ratio of the probability of premature death with ozone exposure to the probability of premature death without ozone exposure; β is the exposure-response coefficient between ozone concentration and premature mortality, indicating the percentage increase in the risk of premature death when ozone concentration rises by 10 μg/m^3^; x represents the ozone concentration in the warm season of a particular year; x_f_ is the threshold value, below which ozone is considered to have no impact on the risk of premature death.

#### 2.3.5. θslope trend analysis method

This study adopts the θslope trend analysis method, which is based on the normalization vegetation index research [32]. The method fits the trend of the population exposure risk index for each pixel over time using a simple linear regression model. The calculation formula is as follows:

$$\theta_{slope}=\frac{n\times \sum_{i=1}^{n}(i\times R_{i})-\sum_{i=1}^{n}i\times \sum_{i=1}^{n}R_{i}}{n\times \sum_{i=1}^{n}i^{2}-{\left(\sum_{i=1}^{n}i\right)}^{2}}$$ (6)

where *θ*_*slope*_ is the interannual variation rate of population exposure risk index for each pixel, and i is the annual sequence number; N is the cumulative number of years; *R*_*i*_ represents the population exposure risk index for the i-th year. Based on the results,*θ*_*slope*_>0 indicates an increasing trend, whereas *θ*_*slope*_<0 indicates a decreasing trend.

## 3. Results and discussion

### 3.1. Spatiotemporal patterns and variations

#### 3.1.1. Temporal change characteristics

**Fig 2:**
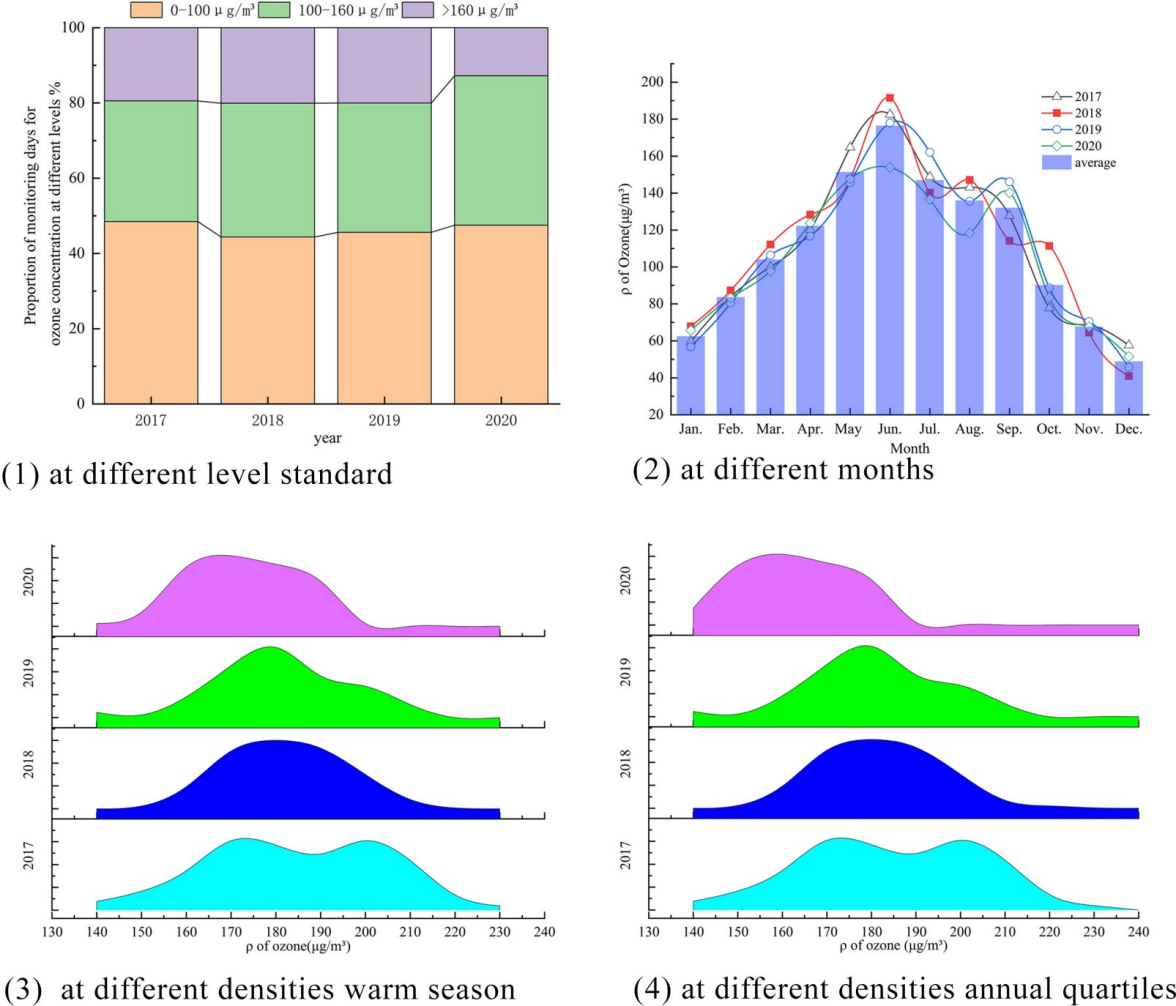
Changes of ozone concentration from 2017 to 2020 in CPUA.

It could be observed that the proportion of ozone concentrations exceeding the national second-level standard (160 μg/m^3^) showed a decreasing trend from 2017 to 2020, with values of 19%, 20%, 20%, and 13% respectively from Fig 2. The concentrations remained relatively stable below the national first-level standard (100 μg/m^3^). However, the proportion of concentrations between the first and second-level standards showed an increasing trend, with values of 32%, 36%, 34%, and 40% respectively. This indicates that overall the ozone emissions of the CPUA have improved to a certain extent.

The trends in ozone concentrations by month show a consistent pattern over the years. The highest concentrations are observed in June, while the lowest concentrations are observed in December. Starting from March, ozone concentrations reach relatively high levels (100 μg/m^3^) and persist until October. This could be attributed to higher temperatures and stronger radiation intensity during this period, which promotes the chemical conversion of ozone precursor substances such as VOCs and NOx.

In terms of peak values, the peak pattern of the CPUA is not stable. From 2017 to 2019, the concentrations followed a bimodal distribution, with peaks in June and secondary peaks in August (2017) or September (2018 and 2019). However, in 2020, a unimodal distribution was observed with the highest peak in June. Considering that most of the CPUA is located north of the traditional boundary line between north and south, which is roughly the Qinling-Huaihe Line, these results are inconsistent with the previous conclusion [16], which showed an unimodal distribution in the north and a bimodal distribution in the south. This suggests that at a micro-level, the relationship between peak values and geographic location is more complex.

From 2017 to 2020, the warm season quartiles (145∼162, 145∼160, 149∼166, 130∼147) and annual quartiles (175∼206, 178∼196, 177∼197, 158∼178) of ozone concentrations at all monitoring stations showed a decreasing trend. However, the values were still higher than the WHO guideline of 60 μg/m^3^ and the national first-level standard of 100 μg/m^3^.

#### 3.1.2. The spatial distribution characteristics

**Fig 3:**
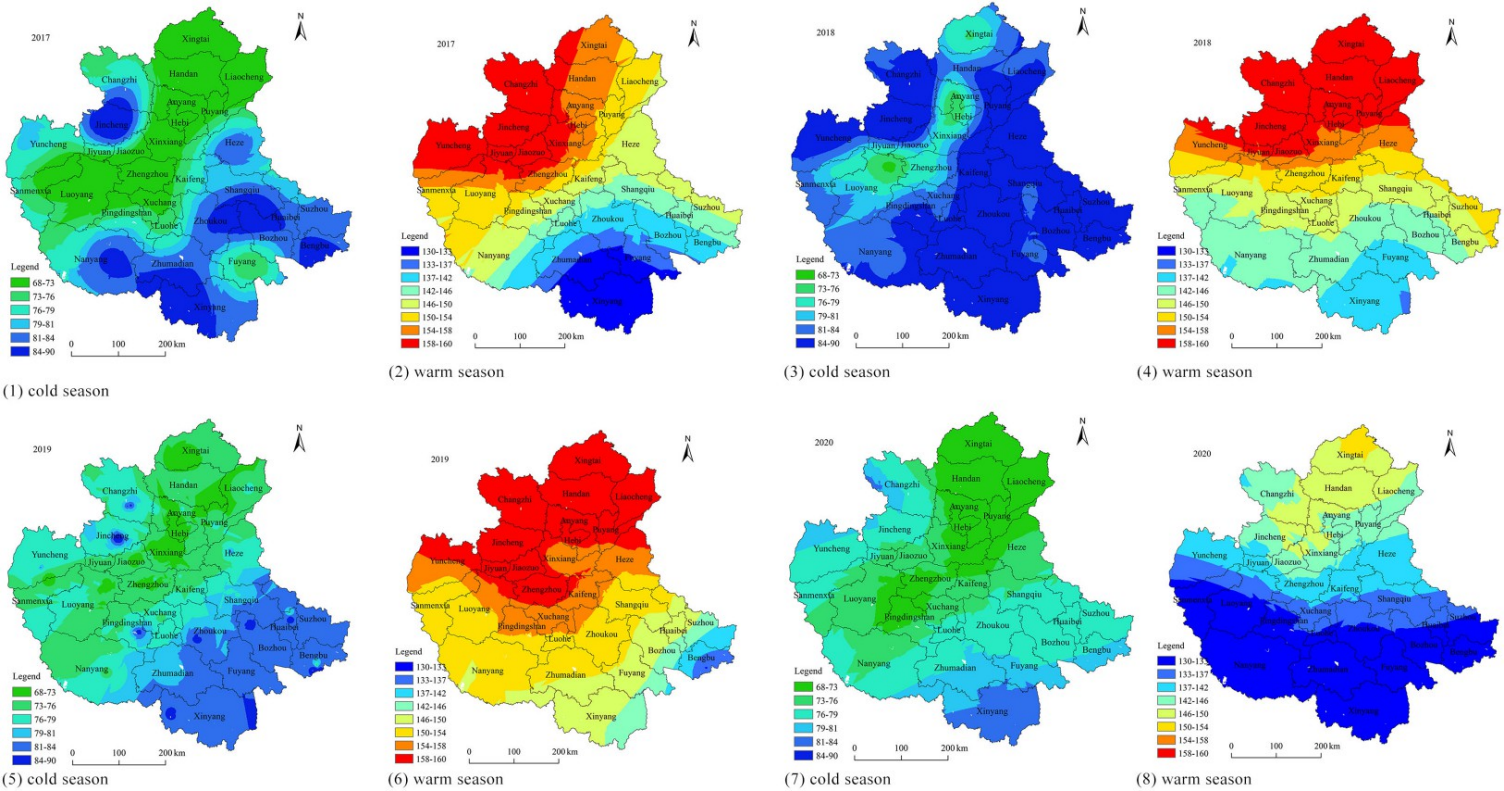
Spatial distribution of ozone concentration from 2017 to 2020.GS (2022)1873.

The spatial distribution characteristics are interpolated based on the Ordinary Kriging method, as shown in Fig 3. The distribution of ozone in the warm season presents a spatial pattern of high in the north and low in the south from 2017 to 2020. The high-value center gradually shifted from Jincheng to Xingtai, while the low-value center shifted from Xinyang to Bengbu. The contour line of 146 μg/m^3^ can effectively reflect the changes in ozone concentration patterns. It extended from Nanyang-Heze in 2017 to south of Xinyang-Suzhou in 2019, and by 2020, it had shifted back north to Changzhi-Xinxiang-Liaocheng.

In the cold season, ozone concentrations ranged from 68 to 90 μg/m^3^. In terms of spatial distribution, it exhibited a high-low-high pattern from northwest to southeast. The low-value region transitioned into the high-value regions on both sides, centered around Xingtai-Hebi-Zhengzhou-Luoyang. The high-value regions were located on both sides of the low-value region, with major high-value centers in Jincheng and Xinyang. The expansion and contraction of the low-value region dominated the changes in the spatial distribution of ozone concentrations from 2017 to 2020. In 2017, the low-value region expanded southward to Luohe, Pingdingshan, eastward to Liaocheng, and westward to Sanmenxia. In 2018, it contracted to Xingtai, Hebi, and Luoyang as the three major low-value centers. From 2019 to 2020, it expanded again throughout the CPUA, with only Xinyang and Changzhi remaining as high-value regions.

Overall, ozone concentrations showed a decrease in both the cold and warm seasons from 2017 to 2020, indicating that the spatial pollution situation is gradually being controlled. Ozone concentrations in the cold season were lower than in the warm season, but the spatial distribution in the cold season was more complex, indicating that factors influencing cold-season ozone pollution may be more complicated. The spatial distribution of ozone in the CPUA during both the cold and warm seasons is still unstable, indicating a variable ozone pollution situation.

### 3.2. Spatial autocorrelation

This study analyzed the spatial clustering of ozone distribution in CPUA using Global Moran’s I, and the results are shown in Table 2. From 2017 to 2020, the Global Moran’s I for ozone concentration was consistently higher than 0.3, with a confidence level of at least 95%, indicating a strong positive spatial autocorrelation and an increasing trend in warm-season ozone concentration.

**Table 2:** Global Moran’s I of ozone concentration in the warm season from 2017 to 2020.

Year	Global Moran’s I	P-value
2017	0.368	0.001***
2018	0.559	0.002***
2019	0.310	0.002***
2020	0.676	0.010***

Note: *,**,***denote respectively 90%、95%、99% confidence level.

To further investigate the spatial clustering of warm-season ozone, Getis-Ord Gi* was employed for hotspot and coldspot clustering analysis, as shown in Fig 3.

**Fig 4:**
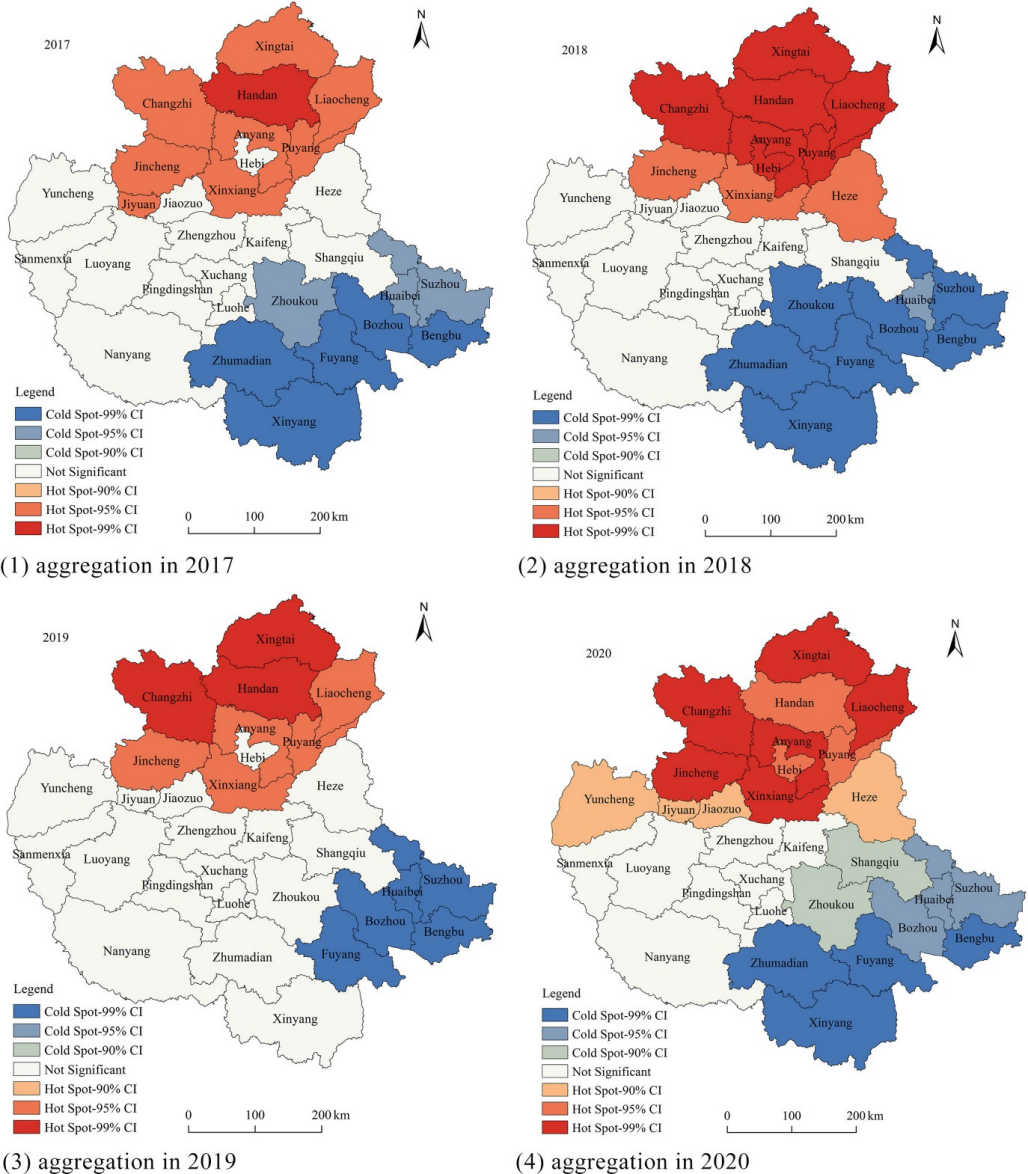
Spatial aggregation of ozone concentration during the warm season from 2017 to 2020.GS (2022)1873.

Along the Sanmenxia-Kaifeng-Nanyang axis, the CPUA can be divided into three parts: the northern region consists mainly of hotspot clusters, the western region is characterized by coldspots, and the eastern region is not significant. This pattern was most typical in 2020. From 2017 to 2020, there were only minor changes in the spatial pattern of hotspots and coldspots. The major hotspot cities during this period were Xingtai, Handan, Liaocheng, and Changzhi, while the major coldspot cities were Xinyang, Fuyang, Bozhou, Huaibei, Suzhou, and Bengbu (Fig 4). In terms of temporal variation, there were more significant hotspots and coldspots in 2018 and 2020, whereas fewer significant hotspots and coldspots were observed in 2017 and 2019, which is consistent with the changes in Moran’s index.

### 3.3. Population exposure risk

#### 3.3.1. Spatial variations in population exposure risk

**Fig 5:**
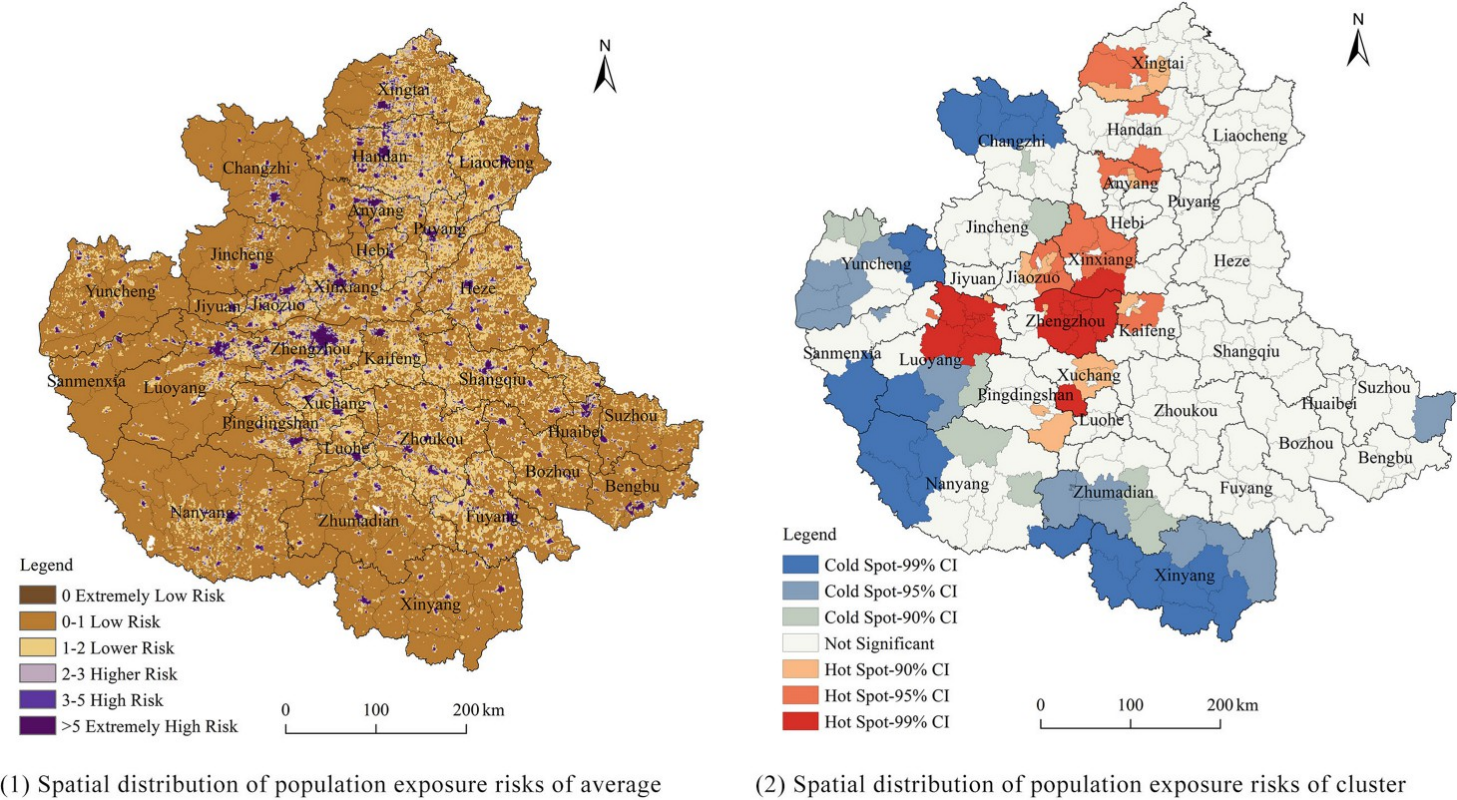
Spatial distribution of population exposure risks.GS (2022)1873.

To study the risk of population exposure in the warm season at a spatial scale, this study quantifies the level of population exposure based on ozone concentration data and population grid data using kriging interpolation. Following the work of Zhang Lianglin et al. [33], the level of population exposure is divided into six categories. *R*_*i*_ = 0 represents extremely low risk; 0 < *R*_*i*_ ≤ 1 represents low risk; 1 < *R*_*i*_ ≤ 2 represents moderate risk; 2 < *R*_*i*_ ≤ 3 represents high risk; 3 < *R*_*i*_ ≤ 5 represents very high risk; and *R*_*i*_ > 5 represents extremely high risk. The average population exposure risk for each county is calculated. The spatial clustering is explored using Global Moran’s I, which reveals a strong positive spatial autocorrelation in the population exposure risk from 2017 to 2020. Furthermore, the specific distribution of hotspots and coldspots is investigated using Getis-Ord Gi*. The spatial distribution and clustering of population exposure risk are shown in Fig 5.

Based on the spatial distribution patterns of ozone exposure in the warm season from 2017 to 2020, the CPUA can be divided into eastern and western parts. The eastern region includes cities such as Changzhi, Jincheng, Yuncheng, Sanmenxia, Luoyang, and Nanyang, while the remaining areas belong to the western region. The terrain in the eastern region is mostly hilly and mountainous, which influences the distribution of risk. The exposure risk is higher in valleys and the central parts of basins. In contrast, the eastern region is mainly composed of plains, and its distribution patterns differ from those in the western region. At the city level, high-risk areas are centered around each city’s jurisdiction and gradually spread outwards, forming cluster-like distributions. At the regional level, cities are connected by a complex transportation network, and high-risk areas extend along this network, forming a mesh-like distribution. For example, the cities of Zhengzhou, Luoyang, Xuchang, and Pingdingshan form a X-shaped network.

**Fig 6:**
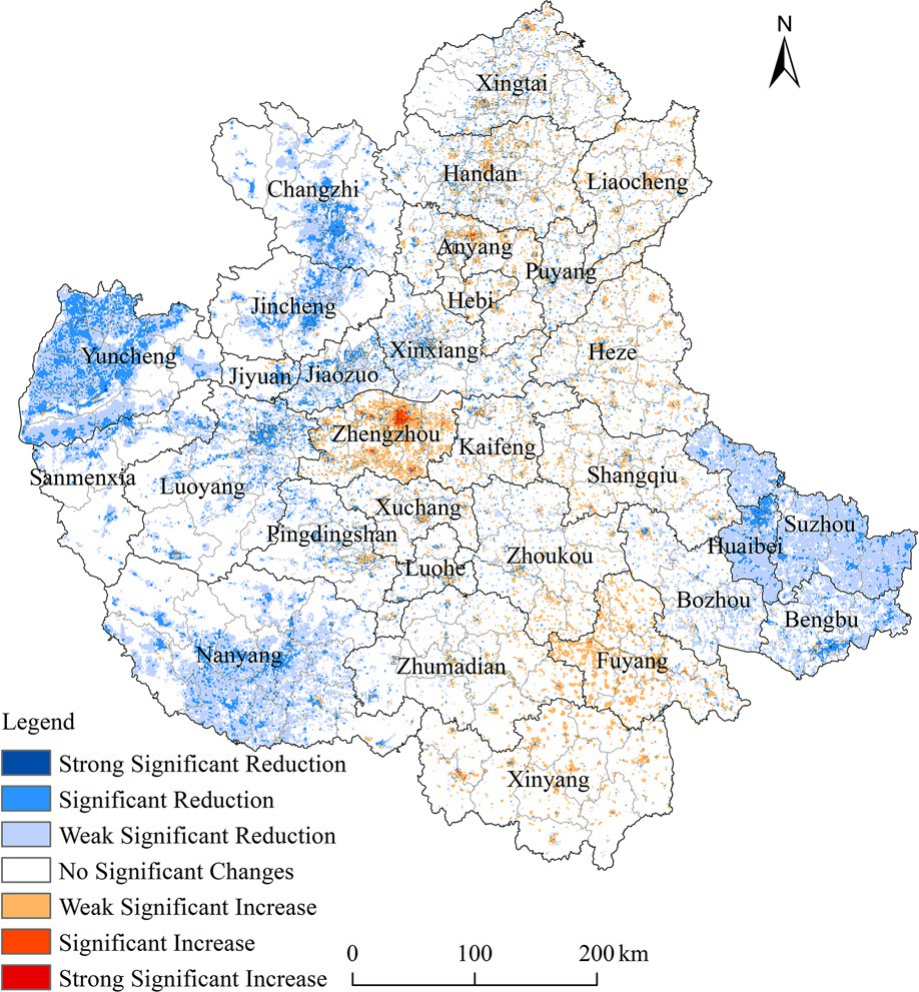
The spatial distribution of average population exposure risk from 2017 to 2020.GS (2022)1873.

In terms of spatial clustering, as shown in the map of Fig 6, the hotspots can be roughly divided into three areas. Firstly, centered around the jurisdiction of Zhengzhou, it extends north and south to Luohe and Xinxiang, with the confidence level gradually decreasing. Secondly, the jurisdiction of Luoyang also exhibits high confidence level, but there is no tendency to spread to other areas. Thirdly, in Anyang, Handan, and Xingtai, there are scattered hotspots with lower confidence levels in certain counties. The coldspots, on the other hand, are mainly distributed in the western hilly and mountainous regions.

#### 3.3.2. Temporal variations in population exposure risk

Using the θslope trend analysis method, the changing trends of seven types of ozone population exposure risks in the CPUA were identified, including significantly increasing, moderately increasing, weakly increasing, no significant change, weakly decreasing, significantly decreasing, and strongly decreasing. It can be observed that from 2017 to 2020, the majority of ozone population exposure risks in the CPUA showed no significant change or a decreasing trend, while a smaller proportion exhibited an increasing trend. The decreasing trend was mainly characterized by weak or significant decreases and was concentrated in the northwestern, western, southwestern, and eastern parts of the agglomeration. The increasing trend was primarily concentrated in Zhengzhou, with some distribution in Xinyang and Fuyang in the south. By applying the normalized vegetation index research-based linear regression method, the temporal trends of population exposure risk indices for each pixel over time were fitted. The results are illustrated in the following Fig 6.

The spatial distribution of areas with increasing and decreasing risks shows a regular pattern. Roughly, the areas between 114°E and 117°E experienced increasing risks, while areas beyond these two meridians experienced decreasing risks. The degree of risk increase or decrease varied across different regions. The risk reduction was more significant in Yuncheng, Jincheng-Changzhi, Luoyang-Jiaozuo, Nanyang, and Huaibei-Suzhou-Bengbu regions, while Zhengzhou and Fuyang saw larger increases in exposure risk. Other regions showed mixed patterns of risk increase or decrease.

### 3.4. Population health effect

**Fig 7:**
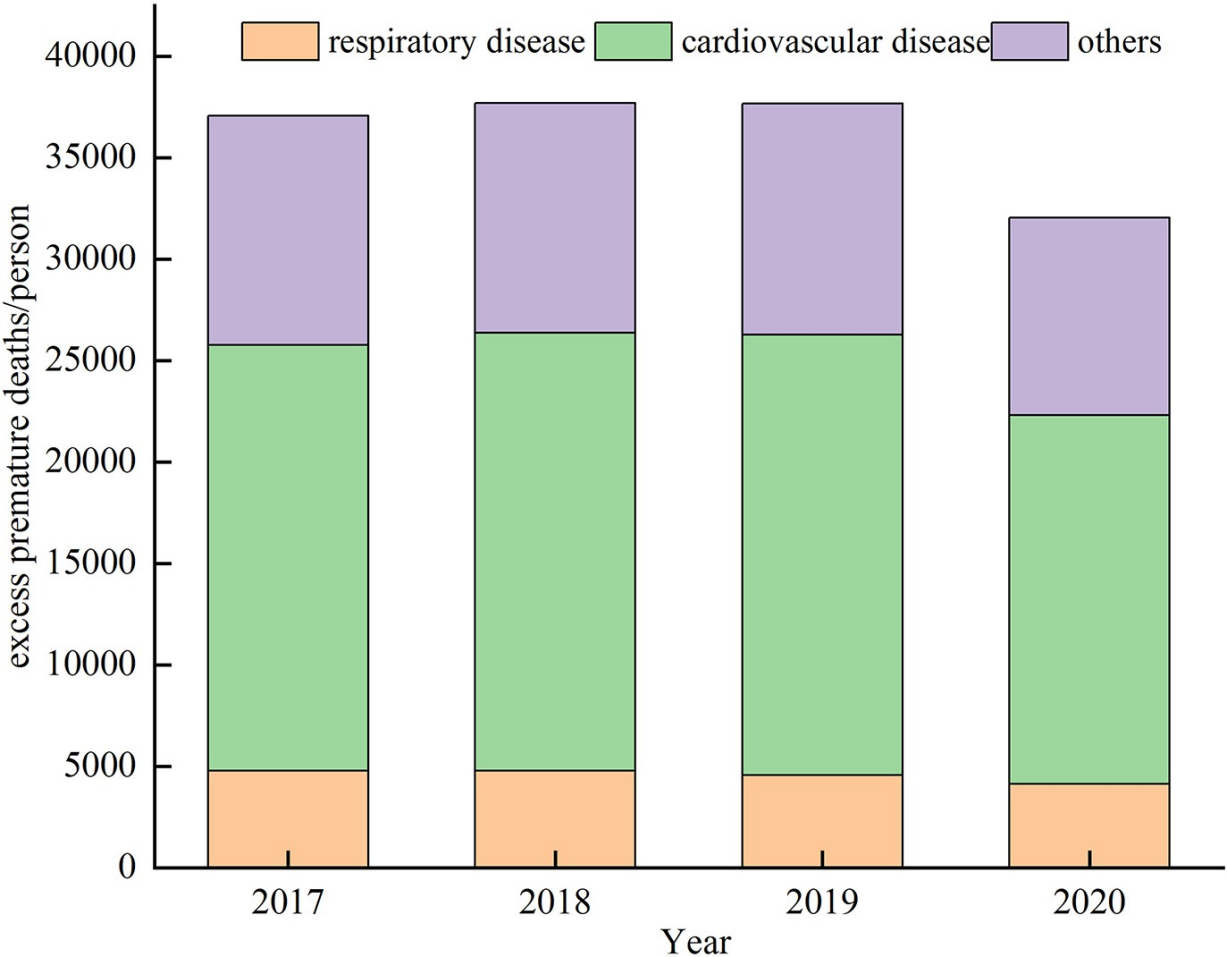
Excess premature deaths attributable to warm season O_3_ pollution in the CPUA from 2017 to 2020.

**Fig 8:**
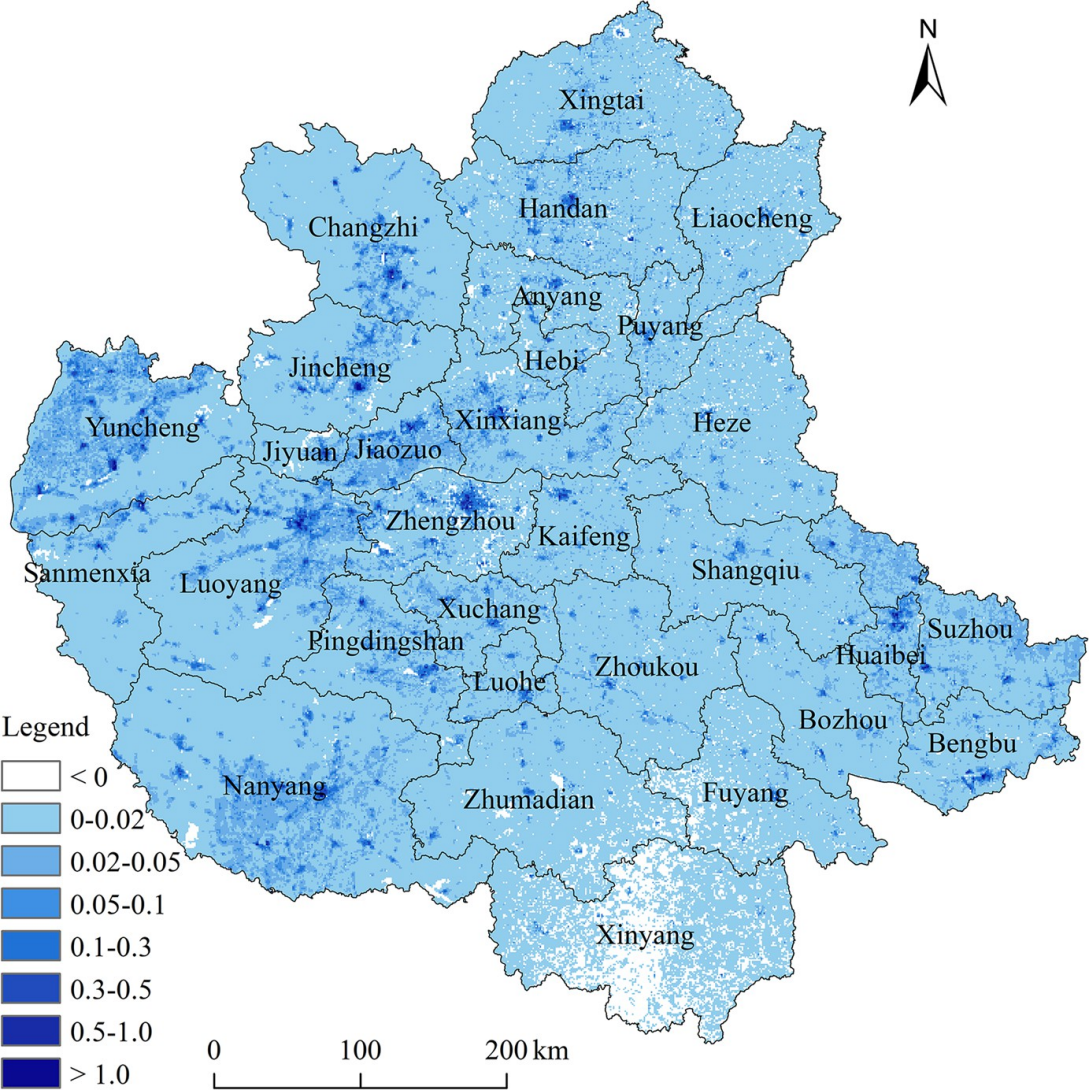
Spatial distribution of avoided premature deaths of improved warm season O_3_ concentrations in 2020 compared to 2017 in the CPUA.GS (2022)1873.

The trend of premature deaths in the CPUA is presented in Fig 8 from 2017 to 2020 due to variations in warm-season ozone concentrations in Fig 7.

It can be seen that the number of premature deaths caused by ozone pollution in the warm season were 37,053 people at 95% confidence interval (95% CI: 28,190–45,930) in 2017, 37,685 people (95% CI: 28,669–46,713) in 2018, and 37,655 people (95% CI: 28,647–46,676) in 2019. The change in excess premature deaths between 2018 and 2019 compared to 2017 was not significant. However, in 2020, the number of excess premature deaths due to warm-season ozone pollution was 32,030 (95% CI: 24,354–39,727), representing a 13.56% decrease compared to 2017. Among various causes of premature deaths, cardiovascular diseases were the primary reason for premature deaths related to ozone pollution exposure. From 2017 to 2020, the improvements in warm season ozone concentrations resulted in an estimated 4,813 avoidable premature deaths (95% CI: 3,649–5,988) in the CPUA, with cardiovascular diseases 621(95% CI:304,937) and respiratory system diseases2738(95% CI:942,3974) accounting for 56.89% and 12.90%.

Here displays the spatial distribution of the health benefits in terms of the avoidable premature deaths in 2020 compared to 2017 in the CPUA (Fig 8). It can be observed that areas with higher population density, such as Zhengzhou, Luoyang, Jiaozuo, and Xinzheng, experienced more significant improvements in ozone concentrations and, consequently, greater health benefits. In the southern part of Henan Province, such as Xinyang and Fuyang, where ozone concentrations increased compared to 2017, positive health benefits were not observed. This reflects the complexity of ozone pollution in the CPUA.

## 4. Conclusions

1. The number of days with severe ozone pollution in the CPUA showed a fluctuating downward trend from 2017 to 2020, with 13% of days exceeding the national secondary standard in 2020. The quartiles of ozone concentrations during the warm season and the whole year at monitoring sites also exhibited a declining trend. The monthly concentration of ozone displayed an unstable pattern, with the main peaks occurring in June, followed by August or September, and the lowest concentration observed in December.
2. The spatial distribution pattern of ozone concentration in the CPUA has undergone significant changes from 2017 to 2020. During the warm season, a higher in the north, lower in the south spatial pattern was observed, with the high-value center shifting from Jincheng to Xingtai and the low-value center shifting from Xinyang to Bengbu. In the cold season, the spatial distribution pattern of ozone concentrations was more complex, showing a high-low-high differentiation pattern from northwest to southeast. The low-value areas in different years were generally distributed along the Xingtai-Hebi-Zhengzhou-Luoyang axis, but the width of these areas varied among different years. Hotspots of the CPUA were concentrated in the northern region, while cold spots were concentrated in the southeast. Xingtai, Handan, and Liaocheng had higher confidence in being hotspots, while Bozhou, Huaibei, Bengbu, and Suzhou had higher confidence in being cold spots.
3. The population exposure risk of ozone in the warm season has obvious spatial distribution characteristics. In the eastern mountainous and hilly regions, it was primarily concentrated in the central parts of valleys and basins. In the western region, it spread outward from the urban districts, forming block-like patterns, with spatial distribution resembling networks in areas with dense transportation networks between cities. Hotspots of risk were identified in Zhengzhou, Luoyang, Anyang, and Xingtai, while cold spots were identified in Changzhi, Yuncheng, Nanyang, and Xinyang.
4. There was a decrease in premature deaths attributable to warm-season ozone in 2020 compared to 2017, with an estimated 4813 avoidable premature deaths (95% CI: 3649–5988). In terms of spatial distribution, except for Xinyang and Fuyang, where premature deaths increased, the number of premature deaths decreased in other areas.

## Supporting information

S1 Table (XLSX)

S2 Table (XLSX)
